# Assessment of multidimensional self-concept in the area of physical education: validation of a scale

**DOI:** 10.3389/fspor.2024.1333751

**Published:** 2024-01-31

**Authors:** Carmen Galán-Arroyo, Santiago Gomez-Paniagua, Antonio Castillo-Paredes, Jorge Rojo-Ramos

**Affiliations:** ^1^ Physical and Health Literacy and Health-Related Quality of Life (PHYQOL), Faculty of Sport Sciences, University of Extremadura, Caceres, Spain; ^2^ BioẼrgon Research Group, Faculty of Sport Sciences, University of Extremadura, Cáceres, Spain; ^3^ Grupo AFySE, Investigación en Actividad Física y Salud Escolar, Escuela de Pedagogía en Educación Física, Facultad de Educación, Universidad de Las Américas, Santiago, Chile; ^4^ Physical Activity for Education, Performance and Health (PAEPH) Research Group, Faculty of Sports Sciences, University of Extremadura, Cáceres, Spain

**Keywords:** self-concept, validation, physical education, secondary school, adolescence

## Abstract

**Introduction:**

Self-concept is a person's perception of him/herself and how he/she performs in different situations. This aspect must be developed during the formative stages and Physical Education is a fundamental area due to its enormous methodological possibilities.

**Aim:**

Therefore, the aim of this study is to evaluate the factor structure and reliability of an instrument that allows to analyze the multidimensional self-concept of high school students in the context of Physical Education.

**Method:**

The sample consisted of 1,155 secondary school students from public schools in the Autonomous Community of Extremadura, Spain. After the exploratory and confirmatory analyses, a structure composed of 30 items divided into 5 factors was.

**Results:**

This design showed excellent goodness-of-fit indices as well as good internal reliability indicators (Cronbach's Alpha = 0.76 – 0.88).

**Conclusion:**

Therefore, this scale can be considered as a tool to assess the self-concept of high school students in a quick and easy way.

## 1 Introduction

The prevalence of mental illness among young people has increased to 20% globally, making it a public health concern (1). In this sense, adolescence is a crucial stage for a person's development because it is during this time that many of the behaviors and routines that become a part of one's lifestyle are established (2). However, insufficient physical activity (PA) is present in 17.8% of boys and 31.9% of girls between the ages of 15 and 24, because their levels tend to fall with age (3). One of the most crucial elements in the prevention and treatment of various kinds of problems is PA (4), being school-based physical education (PE) one of the most accessible forms of PA for kids and adolescents (5). In general terms, PA produces an increase in executive function (6), psychological well-being (7), body satisfaction (8), and self-concept (9), as well as a decrease in depression and anxiety (10), have all been linked to PA in studies on the subject of mental health (11). In fact, given that it has a broad impact on mental health, psychological well-being, and behavior, self-concept is particularly important during the school years and is regarded as a major educational goal (12).

Self-concept has been defined as the individual's perceptions of himself, which are based on his experiences with others and the attributions he makes of his own behavior (13), as well as the individual's concept of himself as a physical, social and spiritual being (14). Under this view, there was a break with the theory prior to the 1980s, advocating a multidimensional structure of self-concept so that any person has a global self-evaluation of himself at the same time that he has more specific self-evaluations (15), differentially related to various areas of human behavior (16). In this context, adolescence is a critical period for the development of self-concept, during which physical, social and emotional changes occur and, therefore, negative self-perceptions may intensify (17), to which must be added the identity crisis that is usually experienced during this stage (18). However, the scientific literature is contradictory, with some studies finding an improvement in self-concept in early adolescence and a decline thereafter (19), while others argue for a U-shaped trend that reaches its lowest level in mid-adolescence (20), although this may be mainly due to the multidimensional nature of self-concept.

Conversely, school PE is acknowledged as a crucial opportunity to increase adolescents' PA mainly due to two perfectly described reasons, the possibility it offers to children and adolescents to accumulate moderate and/or vigorous PA (21) as well as providing them with motor skills, knowledge and a positive attitude towards PA (22). In addition, PA has recently been recognized as a potential marker of health among adolescents (23). In this sense, schools are urged to make sure that physical activity (PA) takes up the bulk of physical education (PE) class time and accounts for a sizable portion of students' daily PA requirements (24), however, most countries only offer a small number of weekly PE lessons, particularly throughout secondary education (25). In addition to the contribution of PE classes on the acquisition of PA habits, these generates an important improvement in self-concept through positive self-evaluation strategies, promoting self-esteem as well as developing students' skills and competences (26, 27).

Similarly, the literature shows numerous scales and questionnaires whose object of analysis is self-concept in adolescence (28, 29). However, and especially in the context of PE, these tools focus on the self-concept that students have at the physical level (30, 31), leaving aside other equally important dimensions such as academic or social, for example. This knowledge gap generates a series of limitations when implementing physical activity-based programs in the classroom that produce improvements in all dimensions of self-concept, because it will not be possible to understand how the socio-demographic characteristics of the students affect their self-concept, making it impossible to specifically design and adapt tasks and activities (32); to know how and to what extent each one of the dimensions of self-concept is related to the practice and acquisition of healthy lifestyle habits (33) and to assess how each of the dimensions is related in the adolescent stage in order to obtain a better self-concept at a general level (34).

In this direction, finding a gap that assesses the constructs of self-image within the physical education classroom, it has been considered important to investigate it for the benefits it could provide to the educational community. Therefore, the aim of this study is to present the psychometric properties, as well as validity and reliability issues of a questionnaire aimed at assessing the self-concept of secondary school students in the context of PE in one of the Autonomous Communities of Spain, Extremadura. In this way, it will be possible to know the current state of the self-concept of secondary school students so that the Ef curriculum can be adapted according to their needs and provide benefits to students at this educational level.

## 2 Methods

### 2.1 Sample

The sample consisted of 1,155 secondary school students from public schools in the Community of Extremadura. A convenience sampling method was used for recruitment. Table 1 shows the sociodemographic characteristics of the participants.

**Table 1:** Sample characterization (*N* = 1,155).

Variable	Categories	*N*	%
Gender	Male	564	48.8
	Female	591	51.2
Grade	First (13 years old)	221	19.1
	Second (14 years old)	213	18.4
	Third (15 years old)	166	14.4
	Fourth (16 years old)	277	24.0
	Fifth (17 years old)	247	21.4
	Sixth (18 years old)	31	2.7
Province of the school	Cáceres	627	54.3
	Badajoz	528	45.7
School environment	Urban	787	68.1
	Rural	368	31.9

*N*, number; %, percentage.

### 2.2 Instruments

First, a sociodemographic questionnaire composed of four questions (gender, grade, province of the school and school environment) was provided. The Spanish Five-Factor Self-Concept Questionnaire (AF-5) (35) was also used to analyze the students' self-concept. This scale is structured in 5 dimensions, each one of them containing 6 items. Dimension 1 (academic self-concept) refers to the student's perception of the quality of his/her role performance, either according to the feedback from his/her teachers or according to the qualities that the student perceives he/she possesses in this context. Dimension 2 (social self-concept), on the other hand, refers to the individual's social network and how easy or difficult it is to maintain and expand it, as well as to some important qualities in interpersonal relationships. The third dimension (emotional self-concept) states general perceptions about his or her general condition and specific situations where another person has a higher rank (teacher). The fourth dimension (family self-concept) analyzes aspects related to parents, family and home. Finally, the fifth dimension (physical self-concept) assesses the student's perceptions of his or her physical appearance and physical condition. The indirect items were swapped before data analysis such that they corresponded to each of the qualities stated above. The responses were based on a Likert scale of 1 (strongly disagree) to 5 (strongly agree). The authors reported an internal consistency value of 0.815 in the original paper, being greater than 0.70 for all tool dimensions (35).

### 2.3 Procedure

The questionnaire was created using the Google Forms tool and included the AF-5 and sociodemographic items. The use of an electronic questionnaire was chosen because it facilitated distribution, saved time, and allowed for the storage of all responses in a single database, increasing the return rate (36).

In order to access the sample, the Department of Education and Employment of the Regional Government of Extremadura's database of public schools in the Autonomous Community of Extremadura (Spain) was used (available at: http: http://estadisticaeducativa.educarex.es/?centros/ensenanzas/&curso=17&ensenanza_centro=101200001, accessed on September 2022). Contact information was chosen for centers offering courses belonging to secondary education (13–18 years). The physical education teachers were then informed about the study and asked to participate through email. The informed consent form was distributed to the schools that expressed interest in taking part, and legal guardians were required to sign it. Then, a researcher visited each educational facility to gather information from the students there. The socio-demographic questionnaire was first completed by the participants in the usual classroom while the researcher and teacher were present. The teacher told the adolescents whether to mark rural or urban depending on the features of their school (previously agreed upon with the researcher based on whether the localities had more or less than 30,000 residents). According to the Diputación de Cáceres website (https://www.dip-caceres.es, visited on September 2022), areas with fewer than 30,000 residents were regarded as rural. Next, the AF-5 scale was provided to the students. The surveys were distributed using tablets that belonged to the study team and were set up for this purpose in order to prevent technical mistakes. The entire data was collected anonymously, and the typical response time was 6 min. Data were collected between October, November, and December 2022.

### 2.4 Statistical analysis

Using a free statistical program called FACTOR v.10.10.02 (Rovira I Virgili University: Tarragona, Spain) (37), the exploratory analyses (EFA) were performed. This program took into account the ordinal nature of the data collected using a 5-choice Likert scale. A robust unweighted least squares (RULS) method with Promin rotation (38) was used for the factor extraction, presuming that there existed a correlation between them (39). The character of the data was taken into account using a polychoric correlation matrix (40), and the proper number of dimensions was established using parallel analysis (41). Once the number of dimensions was established, a normalized weighted oblimin (42) was selected as the rotation technique for determining factor simplicity and structure. Also, the Kaiser–Meyer–Olkin (KMO) and Bartlett tests of sphericity were used as sampling adequacy measures (43).

The AMOS v.26.0.0 software program (IBM Corporation, Wexford, PA, USA) was then used to do the confirmatory factor analysis (CFA). The components with loads lower than 0.60, crossloads greater than 0.40, and communalities lower than 0.30 were eliminated (44). Indicators were employed to gauge the model's goodness-of-fit, including: a root mean square error of approximation (RMSEA) (45), a root mean square of residuals (RMSR) (46), a chi-square per degree of freedom ratio (CMIN/DF) (47), the required non-significant values (*p* > 0.05) for the chi-squared probability calculation (48), a non-normed fit index (NFI) (49), and a comparative fit index (CFI) (50). Additionally, reliability indices such as Cronbach's alpha and McDonald's omega were employed to assess the questionnaire's final design (51).

## 3 Results

Using an RULS technique (52) with Promin rotation in the first half of the sample, five components linked to explained variance based on eigenvalues (53) and the validity of expected a posteriori (EAP) scores (54) were supplied. The sample adequacy indexes produced positive results (Bartlett test = 6518.2; df = 435; *p* = 0.000; and KMO test = 0.86769), which led to the execution of the EFAs. A weighted oblimin rotation method was selected once the number of dimensions was established since the quantity of kurtosis (kurtosis = 41.712; *p* = 0.000) called for non-parametric methods. The rotational loading matrix for 30 items and 5 factors is shown in Table 2.

**Table 2:** Loading matrix.

Items	Family	Social	Physical	Emotional	Academic
1. I do my schoolwork well	−0.003	0.080	−0.063	0.028	0.778
2. I make friends easily	−0.024	0.956	−0.063	−0.018	−0.045
3. I am afraid of some things	−0.061	−0.035	−0.129	0.612	0.062
4. I am criticized a lot at home	0.744	−0.081	−0.039	−0.099	0.004
5. I take care of myself physically	−0.060	−0.093	0.717	0.073	0.101
6. My teachers consider me a good student	0.004	−0.027	−0.034	0.045	0.895
7. I am a friendly person	0.027	0.789	−0.211	0.081	0.144
8. Many things make me nervous	−0.067	0.030	−0.019	0.700	−0.066
9. I am happy at home	0.814	0.116	−0.008	0.006	0.038
10. I am sought after for sports activities	−0.134	0.273	0.435	−0.097	0.061
11. I work hard in class	0.050	−0.056	−0.042	0.032	0.762
12. It is difficult for me to make friends	0.041	0.768	−0.151	−0.087	0.001
13. I am easily frightened	−0.025	−0.084	−0.145	0.553	0.079
14. My family is disappointed with me	0.568	−0.025	0.029	−0.070	0.281
15. I consider myself elegant	0.012	0.224	0.347	0.131	0.111
16. My teachers esteem me	0.018	0.144	0.011	0.024	0.582
17. I am a cheerful person	0.294	0.595	−0.031	0.022	0.032
18. When my elders tell me something I get very nervous	−0.127	−0.112	0.061	0.598	0.061
19. My family would help me in any kind of problems	0.843	0.063	0.113	0.039	−0.026
20. I like the way I am physically	0.247	0.010	0.565	−0.013	−0.005
21. I am a good student	0.005	0.010	−0.026	0.034	0.922
22. I find it hard to talk to strangers	−0.236	0.457	−0.174	0.041	−0.042
23. I get nervous when asked by the teacher	−0.002	−0.065	0.004	0.580	−0.068
24. My parents give me confidence	0.815	−0.032	0.108	0.024	0.020
25. I am good at sports	−0.102	0.132	0.649	−0.121	−0.021
26. My teachers consider me smart and hardworking	−0.010	0.002	0.051	−0.060	0.848
27. I have many friends	0.036	0.697	0.103	−0.005	0.041
28. I feel nervous	−0.146	−0.068	0.028	0.653	−0.018
29. I feel loved by my parents	0.840	−0.040	0.229	0.025	0.033
30. I am an attractive person	0.180	0.243	0.401	0.145	0.001

Table 3 displays each item's structure and factor loadings (Spanish version can be found in Appendix B). The five correlated factors in the factor solution were as follows: (1) Academic self-concept; (2) Social self-concept; (3) Emotional self-concept; (4) Family self-concept and (5) Physical self-concept.

**Table 3:** Factor loading and factor solution.

Items	Family	Social	Physical	Emotional	Academic
1. I do my schoolwork well					0.778
2. I make friends easily		0.956			
3. I am afraid of some things				0.612	
4. I am criticized a lot at home	0.744				
5. I take care of myself physically			0.717		
6. My teachers consider me a good student					0.895
7. I am a friendly person		0.789			
8. Many things make me nervous				0.700	
9. I am happy at home	0.814				
10. I am sought after for sports activities			0.435		
11. I work hard in class					0.762
12. It is difficult for me to make friends		0.768			
13. I am easily frightened				0.553	
14. My family is disappointed with me	0.568				
15. I consider myself elegant			0.347		
16. My teachers esteem me					0.582
17. I am a cheerful person		0.595			
18. When my elders tell me something I get very nervous				0.598	
19. My family would help me in any kind of problems	0.843				
20. I like the way I am physically			0.565		
21. I am a good student					0.922
22. I find it hard to talk to strangers		0.457			
23. I get nervous when asked by the teacher				0.580	
24. My parents give me confidence	0.815				
25. I am good at sports			0.649		
26. My teachers consider me smart and hardworking					0.848
27. I have many friends		0.697			
28. I feel nervous				0.653	
29. I feel loved by my parents	0.840				
30. I am an attractive person			0.401		

The relationship between the five dimensions of the AF-5 questionnaire—Academic self-concept, Social self-concept, Emotional self-concept, Family self-concept and Physical self-concept—is shown in Table 4.

**Table 4:** Correlations between scale dimensions.

	Factor 1 Academic self-concept	Factor 2 Social self-concept	Factor 3 Emotional self-concept	Factor 4 Family self-concept	Factor 5 Physical self-concept
Factor 1 Academic self-concept	1				
Factor 2 Social self-concept	0.136	1			
Factor 3 Emotional self-concept	0.018	−0.245	1		
Factor 4 Family self-concept	0.401	0.252	−0.218	1	
Factor 5 Physical self-concept	0.213	0.572	−0.212	0.265	1

**Figure 1:**
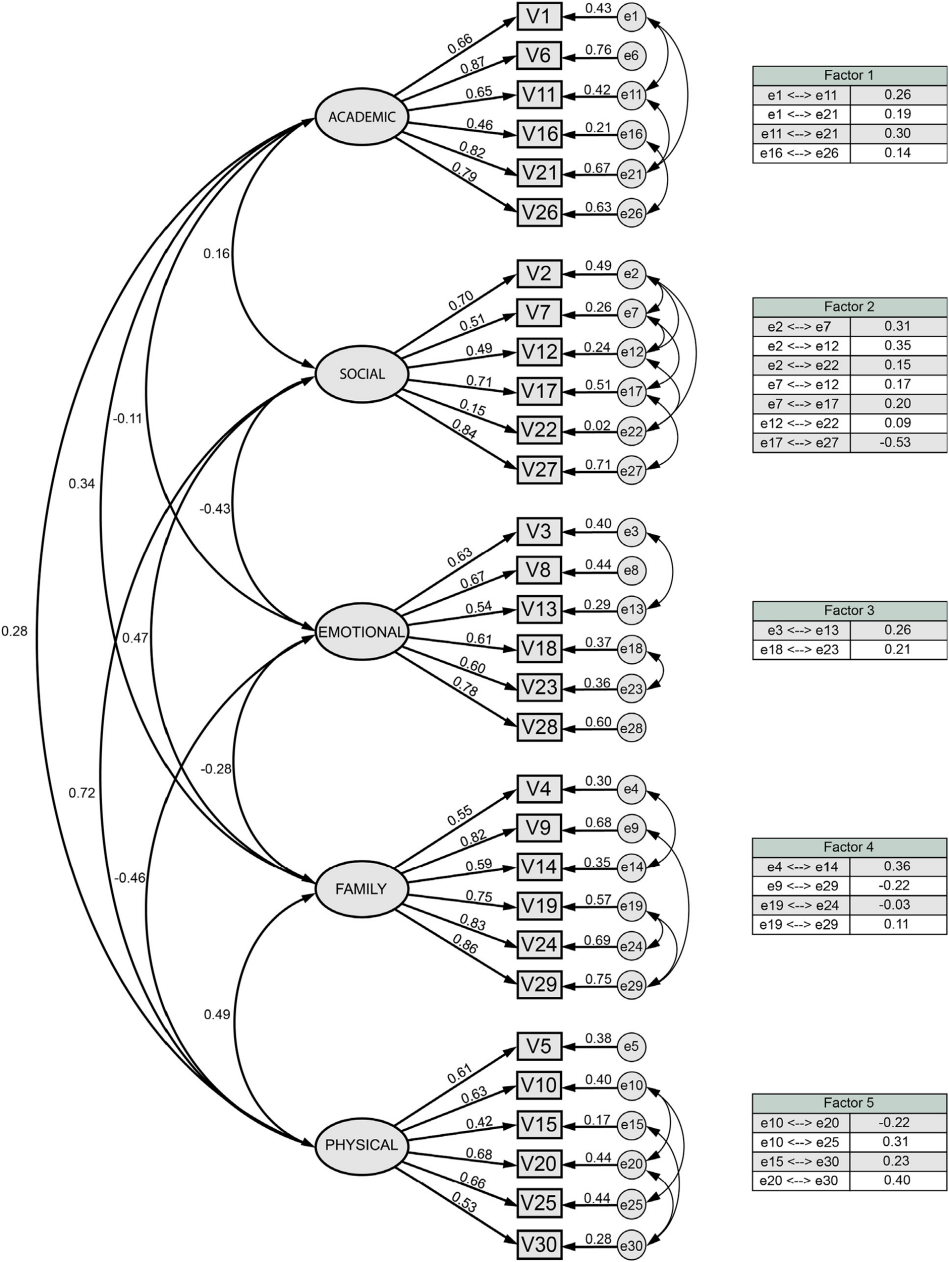
Factor model of the AF-5.

Following the definition of the questionnaire's structure, a CFA was conducted with the remaining half of the sample to create a conclusive model (Figure 1).

Figure 1 shows the questionnaire's final format, which consists of 30 items separated into 5 dimensions and lists the following values from left to right: (1) correlations between factors, (2) normalized regression weights, (3) squared multiple correlations of each variable, and (4) correlations between exogenous variables (tables).

Table 5 displays the AF-5 goodness-of-fit indices that were obtained after the CFA, demonstrating each one of them a strong fit between the data and the model (55). The RMSEA was within the permitted range (0.010–0.050), and the RMSR, at less than 0.08, qualifies as accurate. Additionally, the non-significant values contributed to the excellent chi-squared probability. The CMIN/DF index also exhibited excellent values given that it must be less than 2 to be an acceptable model fit. Finally, NFI and CFI values greater than 0.9 demonstrated a good fit to the model.

**Table 5:** Af-5 goodness-of-fit indicators.

Indices	Value
RMSEA	0.041
RMSR	0.062
*Ρ (χ^2^)*	0.981
CMIN/DF	1.970
NFI	0.904
CFI	0.950

Table 6 shows the reliability indices for the AF-5 questionnaire dimensions using Cronbach's alpha, McDonald's omega, and the explained variance of each factor.

**Table 6:** Internal consistency of the scale.

	Factor 1 Academic self-concept	Factor 2 Social self-concept	Factor 3 Emotional self-concept	Factor 4 Family self-concept	Factor 5 Physical self-concept
Cronbach’s Alpha	0.877	0.763	0.805	0.878	0.764
McDonald's Omega	0.844	0.780	0.806	0.878	0.768
Explained variance	4.196	3.553	2.520	4.284	2.081

For each of the components, the Cronbach's alpha and McDonald's omega scores were satisfactory because they were higher than 0.7 (56). The explained variance is the proportion of the variance in the responses that was not attributable to hazard (residual values) but was instead assigned to each of the model's components.

## 4 Discussion

The main contribution of this study is the assessment of the psychometric properties of the AF-5 scale that allows the evaluation of general self-concept and its dimensions specifically within the context of PE in secondary school students. Similarly, reliability and validity issues were analyzed so that the application of the instrument could be repeated in future research studies focusing on this population. The results yield a configuration of 30 items encompassed in 5 interrelated dimensions, all of them with excellent goodness-of-fit indices. In addition, reliability indicators showed great values. Originally, the validation and initial development of this scale took place in the Spanish population, to be later translated and adapted to different dialects and languages (35). In this sense, Malo-Cerrato (57) adapted and validated this scale to Catalan, evaluating its psychometric properties in a population sample from 11 to 63 years old, and although they eliminated one factor for convenience, the results of reliability and validity were satisfactory. Likewise, García and colleagues (58) adapted and validated the English version of the AF-5 in a sample of 600 American adolescents with results very similar to the original Spanish version. Later, Garcia et al. (59). adapted the scale again but to the Portuguese language, collecting a large sample of Brazilian adolescent participants and showing excellent results in each of the dimensions of the questionnaire. In addition, validations have been carried out in university populations in Spanish-speaking countries belonging to the South American continent (60, 61). However, to the authors’ knowledge, the AF-5 questionnaire has not been specifically validated in a secondary school population in Spain, much less in the context of PE.

Academic self-concept is the dimension understood as the attitude that conditions behavior and performance in the academic context, related to motivation and determining the behavior to be followed in the face of failure (62), depending not only on the academic achievement of each student, but also on the achievement of everyone in the school (63). Overall, research indicates that higher self-concept scores lead to better academic achievement and other desirable educational outcomes such as confidence and other related academic skills (64). This aspect is crucial because it would help teachers and others involved in the training process to better understand how students' self-concepts are formed and consolidated, enabling them to comprehend students better and give more appropriate feedback, especially to students who are less capable (65). As early as the 1990s, Marsh (66) observed that the subjects of Physical Education and Art were more closely related to dimensions of self-concept other than academic self-concept, such as physical or social. However, Goñi and Zulaika demonstrated that specific pedagogical strategies in the PE classroom can improve students' academic self-concept (67). Therefore, current research is focused on developing programs in the context of PE to promote the improvement of students' academic self-concept (68).

As for social self-concept, researchers have long recognized that self-evaluations are formed in relation to social and temporal comparisons, the better the performance in relation to others and in relation to their past accomplishments the more positive an individual's self-evaluations are (69). Thus, social self-concept is the social creation shaped by a person's interactions with others, his or her past and current afflictions and experiences within social and institutional contexts, and his or her location within the culture and social structure (70). In this context, Baena–Extremera found associations between the improvement of general self-concept and social relations in students participating in adventure programs in PE classes (71). Likewise, establishing a fearless atmosphere to increase students' mutual social acceptance and reduce indirect negative peer comments is a proven strategy in PE classrooms (72). Finally, another study highlights the importance of gender differences, with women perceiving less favorable climates than men and with the most unfavorable perceptions being held by women who have only sisters or no brothers, thus necessitating sports interventions that favor women's participation (73).

Emotional self-concept is related to the person's perception of his or her emotional state and the responses he or she gives to situations in his or her daily life (35). This self-concept also includes motivation, personality development and, in general, the social relationships and affective contact that the person has with him/herself (74). Similar to how behaviors that cannot be separated from an educational setting are heavily influenced by an individual's cultural heritage, emotions are as well (75). Empirical evidence indicates that low scores in this dimension tend to make people more prone to experience anxiety, depression and low performance in activities in general (76). Gómez–Mármol has already demonstrated the great possibilities available to PE teachers to design activities that improve students' emotional expression (77). Similarly, the area of PE has been pointed out as a context of innumerable possibilities to improve the emotional self-concept of students due to the diverse methodologies to propose motor games (78).

The family self-concept dimension focuses on a person's appreciation of relationships, involvement and implications in the family environment (35). High scores on this dimension indicate that people are more adaptive, more stable and their interpersonal relationships are stronger; low scores are associated with imbalance, immaturity and maladjustment (76). However, other authors, such as Loayza Gonzales (62), consider that social and family self-concept should form a single category. Amado-Alonso and collaborators proposed an intervention program based on organized sports practice, finding improvements in the family self-concept of students in the last cycle of primary education (79). Also, another study pointed out that family self-concept was the one that showed the best scores in adolescents who practiced basketball and that the longer the hours of practice, the better the results (80).

Finally, regarding physical self-concept, there is evidence of great theoretical and empirical development, where ability and physical appearance are determinants (81). In general terms, this dimension is relevant, because in adolescence it plays a fundamental role in the conception of self, specifically in the formation of self-image (76). Fernández-Bustos explored generated a theoretical model in adolescents with great results in which PA was positively related to self-concept and the mediators are family self-concept and body image (33). In order to strengthen students’ physical self-concept, it is critical that the subject of physical education considers experiences that support students' sense of personal identity and self-worth through the enhancement of quality motivation (82). Moreover, Spanish primary school students have a better physical self-concept than secondary school students, so that PE teachers at this stage should promote pedagogical strategies to ensure greater adherence to sports practice (83).

### 4.1 Limitations and future lines

The present study has a series of limitations like any other, for example, the analysis is focused on a specific autonomous community (Extremadura), so there are several oscillating demographic and cultural variables that affect the responses of the participants. Likewise, no randomization of the sample was carried out; instead, convenience sampling was used. In addition, the validation of an instrument requires more time and involves many different methodologies and large samples. Finally, the responses were collected through online questionnaires and not through a face-to-face interview method, so the results may vary to a lesser or greater extent. As future lines of research, it is suggested that larger samples from other autonomous communities be collected to validate the instrument throughout Spain. In the same way, results should be compared with other educational levels, such as primary and/or university education, in order to analyze how self-concept evolves throughout various stages of the life cycle.

## 5 Conclusions

This research presents the validity and reliability of a tool to assess the self-concept of secondary school students in the context of the PE area.

Students' self-concept about themselves reveals valuable information about their educational needs, which is even more important in a subject full of possibilities for developing and implementing pedagogical strategies to improve it. Knowing the validity and reliability of the instrument that measures the different dimensions of self-image allows educational authorities, teachers, parents and the whole school community to establish the basis of the construction and to be able to evaluate the tools and methodologies that can be implemented to improve the development of self-image in secondary school physical education students and to reduce the bad mental consequences that follow a poor self-concept in adolescent development.

## Data Availability

The raw data supporting the conclusions of this article will be made available by the authors, without undue reservation.

## References

[1] Belfer ML. Child and adolescent mental disorders: the magnitude of the problem across the globe. J Child Psychol Psychiat. (2008) 49:226–36. 10.1111/j.1469-7610.2007.01855.x 18221350

[2] Bogin B. Adolescence in evolutionary perspective. Acta Paediatr. (1994) 83:29–35. 10.1111/j.1651-2227.1994.tb13418.x 7734808

[3] *Ministerio de Sanidad Encuesta Europea de Salud En España*. Available at: https://www.sanidad.gob.es/estadEstudios/estadisticas/EncuestaEuropea/Enc_Eur_Salud_en_Esp_2020.htm (Accessed on 23 January 2023).

[4] Ahn S Fedewa AL. A meta-analysis of the relationship between children’s physical activity and mental health. J Pediatr Psychol. (2011) 36:385–97. 10.1093/jpepsy/jsq107 21227908

[5] Beasley EK Garn AC. An investigation of adolescent girls’ global self-concept, physical self-concept, identified regulation, and leisure-time physical activity in physical education. J Teach Phys Educ. (2013) 32:237–52. 10.1123/jtpe.32.3.237

[6] Xue Y Yang Y Huang T. Effects of chronic exercise interventions on executive function among children and adolescents: a systematic review with meta-analysis. Br J Sports Med. (2019) 53:1397–404. 10.1136/bjsports-2018-099825 30737201

[7] McMahon EM Corcoran P O’Regan G Keeley H Cannon M Carli V Physical activity in European adolescents and associations with anxiety, depression and well-being. Eur Child Adolesc Psychiatry. (2017) 26:111–22. 10.1007/s00787-016-0875-9 27277894

[8] Hartman-Munick SM Gordon AR Guss C. Adolescent body image: influencing factors and the clinician’s role. Curr Opin Pediatr. (2020) 32:455–60. 10.1097/MOP.0000000000000910 32487854

[9] Garn AC Morin AJS White RL Owen KB Donley W Lonsdale C. Moderate-to-vigorous physical activity as a predictor of changes in physical self-concept in adolescents. Health Psychol. (2020) 39:190–8. 10.1037/hea0000815 31750675

[10] Penedo FJ Dahn JR. Exercise and well-being: a review of mental and physical health benefits associated with physical activity. Curr Opin Psychiatry. (2005) 18:189–93. 10.1097/00001504-200503000-00013 16639173

[11] Brown HE Pearson N Braithwaite RE Brown WJ Biddle SJH. Physical activity interventions and depression in children and adolescents: a systematic review and meta-analysis. Sports Med. (2013) 43:195–206. 10.1007/s40279-012-0015-8 23329611

[12] Viholainen H Aro T Purtsi J Tolvanen A Cantell M. Adolescents’ school-related self-concept mediates motor skills and psychosocial well-being. Br J Educ Psychol. (2014) 84:268–80. 10.1111/bjep.12023 24829120

[13] Shavelson RJ Hubner JJ Stanton GC. Self-concept: validation of construct interpretations. Rev Educ Res. (1976) 46:407–41. 10.3102/00346543046003407

[14] Musitu G Román J. Autoconcepto: una introducción a esta Variable Intermedia. Revista de Psicología, Pedagogía y Filosofía. (1982) 4:51–69. ISSN: 1136-1034. Available at: https://www.redalyc.org/articulo.oa?id=17513105 (Accessed September 10, 2023).

[15] Tomás JM Oliver A. Análisis psicométrico confirmatorio de una medida multidimensional Del autoconcepto en Español. (confirmatory factor analysis of a Spanish multidimensional scale of self-concept). Revista Interamericana de Psicología. (2004) 38:285–93. ISSN: 0034-9690. Available at: https://www.redalyc.org/articulo.oa?id=28438214 (Accessed September 11, 2023).

[16] Goñi Palacios E Fernández Zabala A. Los dominios social y personal del autoconcepto. Soc Pers Domains Self-Concept. (2007). 10/7098

[17] Harter S. The Construction of the Self: A Developmental Perspective; Distinguished Contributions in Psychology. New York: Guilford Press (1999); ISBN 978-1-57230-432-1.

[18] Risoto MA. Influencia del rendimiento y autoconcepto en hombres y mujeres. Revista Electrónica de Investigación y Docencia (REID). (2009). Available at: http://www.revistareid.net/revista/n2/REID2art2.pdf (Accessed September 10, 2023).

[19] Parker AK. A longitudinal investigation of young adolescents’ self-concepts in the middle grades. RMLE Online. (2010) 33:1–13. 10.1080/19404476.2010.11462073

[20] Marsh HW Ayotte V. Do multiple dimensions of self-concept become more differentiated with age? The differential distinctiveness hypothesis. J Educ Psychol. (2003) 95:687–706. 10.1037/0022-0663.95.4.687

[21] Lonsdale C Rosenkranz RR Peralta LR Bennie A Fahey P Lubans DR. A systematic review and meta-analysis of interventions designed to increase moderate-to-vigorous physical activity in school physical education lessons. Prev Med. (2013) 56:152–61. 10.1016/j.ypmed.2012.12.004 23246641

[22] Hills AP Dengel DR Lubans DR. Supporting public health priorities: recommendations for physical education and physical activity promotion in schools. Prog Cardiovasc Dis. (2015) 57:368–74. 10.1016/j.pcad.2014.09.010 25269062

[23] Poitras VJ Gray CE Borghese MM Carson V Chaput J-P Janssen I Systematic review of the relationships between objectively measured physical activity and health indicators in school-aged children and youth. Appl Physiol Nutr Metab*.* (2016) 41:S197–239. 10.1139/apnm-2015-0663 27306431

[24] Harris J. Association for physical education health position paper [2015]. (2017).

[25] Hardman K Routen A Tones S. UNESCO-NWCPEA: world-wide survey of school physical education. Final Report. Paris (2014); ISBN 978-92-3-100048-5.

[26] Stiller J Alfermann D. Promotion of a healthy self-concept. In: Teoksessa Liukkonen J Vanden Auweele Y Vereijken B Alfermann D Theodorakis Y (toim.). Psychology for Physical Educators. Student in Focus. Champaign, IL: Human Kinetics (2007). p. 123–40.

[27] Ferrer-Caja E Weiss MR. Predictors of intrinsic motivation among adolescent students in physical education. Res Q Exerc Sport. (2000) 71:267–79. 10.1080/02701367.2000.10608907 10999264

[28] Saraswat RK. Manual for self concept questionnaire. Agra: National Psychological Corporation (1984).

[29] Benjamin LS. A clinician-friendly version of the interpersonal circumplex: structural analysis of social behavior (SASB). J Pers Assess. (1996) 66:248–66. 10.1207/s15327752jpa6602_5 8869570

[30] Fox KR Corbin CB. The physical self-perception profile: development and preliminary validation. J Sport Exerc Psychol. (1989) 11:408–30. 10.1123/jsep.11.4.408

[31] Marsh HW. Physical self description questionnaire: stability and discriminant validity. Res Q Exerc Sport. (1996) 67:249–64. 10.1080/02701367.1996.10607952 8888413

[32] Grain RM Bracken BA. Age, race, and gender differences in child and adolescent self-concept: evidence from a behavioral-acquisition, context-dependent model. School Psych Rev. (1994) 23:496–511. 10.1080/02796015.1994.12085728

[33] Fernández-Bustos JG Infantes-Paniagua Á Cuevas R Contreras OR. Effect of physical activity on self-concept: theoretical model on the mediation of body image and physical self-concept in adolescents. Front Psychol*.* (2019) 10:1537. 10.3389/fpsyg.2019.01537 31354570 PMC6635469

[34] Marsh HW O’Neill R. Self description questionnaire III: the construct validity of multidimensional self-concept ratings by late adolescents. J Educ Meas. (1984) 21:153–74. 10.1111/j.1745-3984.1984.tb00227.x

[35] García Pérez JF Musitu Ochoa G. AF5, Autoconcepto forma 5: manual, 2nd Edn. Madrid: TEA (2001); ISBN 978-84-7174-677-1.

[36] Anderson T Kanuka H. E-research: methods, strategies, and issues. In: Nachdr. Boston, MA, Munich: Allyn and Bacon (2003); ISBN 978-0-205-34382-9.

[37] Ferrando PJ Lorenzo-Seva U. Program FACTOR at 10: origins, development and future directions. Psicothema. (2017) 29:236–40. 10.7334/psicothema2016.304 28438248

[38] Lorenzo-Seva U Ferrando PJ. Robust promin: a method for diagonally weighted factor rotation. Liberabit. (2019) 25:99–106. 10.24265/liberabit.2019.v25n1.08

[39] McDonald RP. Factor Analysis and Related Methods. New York: Psychology Press (2014); ISBN 978-1-317-76877-7.

[40] Holgado–Tello FP Chacón–Moscoso S Barbero–García I Vila–Abad E. Polychoric versus Pearson correlations in exploratory and confirmatory factor analysis of ordinal variables. Qual Quant. (2010) 44:153–66. 10.1007/s11135-008-9190-y

[41] Hayton JC Allen DG Scarpello V. Factor retention decisions in exploratory factor analysis: a tutorial on parallel analysis. Organ Res Methods. (2004) 7:191–205. 10.1177/1094428104263675

[42] Lorenzo-Seva U. The weighted oblimin rotation. Psychometrika. (2000) 65:301–18. 10.1007/BF02296148

[43] Williams B Onsman A Brown T. Exploratory factor analysis: a five-step guide for novices. Australas J Paramedicine. (2010) 8:1–13. 10.33151/ajp.8.3.93

[44] Brown TA. Confirmatory Factor Analysis for Applied Research; Methodology in the Social Sciences, 2nd Edn. New York, London: The Guilford Press (2015); ISBN 978-1-4625-1779-4.

[45] Kenny DA Kaniskan B McCoach DB. The performance of RMSEA in models with small degrees of freedom. Sociol Methods Res. (2015) 44:486–507. 10.1177/0049124114543236

[46] Shi D Maydeu-Olivares A DiStefano C. The relationship between the standardized root mean square residual and model misspecification in factor analysis models. Multivariate Behav Res. (2018) 53:676–94. 10.1080/00273171.2018.1476221 30596259

[47] Yaşlioğlu M Toplu Yaşlioğlu D. How and when to use which fit indices? A practical and critical review of the methodology. IMJ. (2020):1–20. 10.26650/imj.2020.88.000132298555

[48] Marcoulides GA. Evaluation of confirmatory factor analytic and structural equation models using goodness-of-fit indices. Psychol Rep. (1990) 67:669–70. 10.2466/pr0.1990.67.2.669

[49] Yadama GN Pandey S. Effect of sample size on goodness-fit of-fit indices in structural equation models. J Soc Serv Res. (1995) 20:49–70. 10.1300/J079v20n03_03

[50] Rigdon EE. CFI versus RMSEA: a comparison of two fit indexes for structural equation modeling. Struct Equ Modeling Multidiscipl J. (1996) 3:369–79. 10.1080/10705519609540052

[51] Ravinder EB Saraswathi DAB. Literature review of cronbachalphacoefficient (Α) and McDonald’s omega coefficient (Ω). Eur J Mol Clin Med. (2020) 7:2943–9. 10.13140/RG.2.2.35489.53603

[52] Schreiber JB. Issues and recommendations for exploratory factor analysis and principal component analysis. Res Soc Adm Pharm. (2021) 17:1004–11. 10.1016/j.sapharm.2020.07.027 33162380

[53] Larsen R Warne RT. Estimating confidence intervals for eigenvalues in exploratory factor analysis. Behav Res Methods. (2010) 42:871–6. 10.3758/BRM.42.3.871 20805609

[54] Ferrando Piera PJ Lorenzo Seva U. A note on improving EAP trait estimation in oblique factor-analytic and item response theory models. Psicológica: Revista de Metodología y Psicología Experimental. (2016) 37:235–47. Available at: https://www.redalyc.org/articulo.oa?id=16946248007 (Accessed September 11, 2023).

[55] Sun J. Assessing goodness of fit in confirmatory factor analysis. Meas Eval Couns Dev. (2005) 37:240–56. 10.1080/07481756.2005.11909764

[56] Nunnally JC Bernstein IH. Psychometric Theory. New York: McGraw-Hill, ©1994 (1994).

[57] Malo Cerrato S Bataller Sallent S Casas Aznar F Gras Pérez ME González Carrasco M. Análisis psicométrico de la escala multidimensional de autoconcepto AF5 en una muestra de adolescentes y adultos de cataluña. Psicothema. (2011) 23:871–8. Available at: https://reunido.uniovi.es/index.php/PST/article/view/9172 (Accessed September 12, 2023). 22047886

[58] García F Gracia E Zeleznova A. Validation of the english version of the five-factor self-concept questionnaire. Psicothema. (2013):549–55. 10.7334/psicothema2013.33 24124791

[59] Garcia F Martínez I Balluerka N Cruise E Garcia OF Serra E. Validation of the five-factor self-concept questionnaire AF5 in Brazil: testing factor structure and measurement invariance across language (Brazilian and Spanish), gender, and age. Front Psychol*.* (2018) 9:2250. 10.3389/fpsyg.2018.02250 30515120 PMC6256062

[60] Montoya Londoño DM Dussán Lubert C Pinilla Sepúlveda VE Puente Ferreras A. Estandarización de la escala de autoconcepto AF5 en estudiantes universitarios colombianos. Ansiedad y Estrés. (2019) 25:118–24. 10.1016/j.anyes.2019.06.001

[61] Carranza Esteban RF Bermúdez-Jaimes ME. Análisis psicométrico de la escala de autoconcepto AF5 de garcía y musitu en estudiantes universitarios de tarapoto (perú). Interdisciplinaria. (2017) 34:459–72.

[62] Gonzales DL. Autoconcepto, una revisión Del constructo. Revista Científica de Acceso Abierto de la Universidad Seminario Evangélico de Lima. (2019) 1:29–33.

[63] Self-Concept theory, research & practice: advances for the new millennium: proceedings of the inaugural international conference. In: Craven RG Marsh HW, Editors. Sydney: University of Western Sydney, SELF Research Centre (2000); ISBN 978-1-86341-877-5.

[64] Marsh HW Martin AJ. Academic self-concept and academic achievement: relations and causal ordering: academic self-concept. Br J Educ Psychol. (2011) 81:59–77. 10.1348/000709910X503501 21391964

[65] Marsh HW Shavelson R. Self-concept: its multifaceted, hierarchical structure. Educ Psychol. (1985) 20:107–23. 10.1207/s15326985ep2003_1

[66] Marsh HW. The structure of academic self-concept: the Marsh/Shavelson model. J Educ Psychol. (1990) 82:623–36. 10.1037/0022-0663.82.4.623

[67] Goñi A Zulaika L. Relationships between physical education classes and the enhancement of fifth grade pupils’ self-concept. Percept Mot Skills. (2000) 91:246–50. 10.2466/pms.2000.91.1.246 11011894

[68] Asogwa UD Ofoegbu TO Eseadi C Ogbonna CS Eskay M Nji GC The effect of a video-guided educational technology intervention on the academic self-concept of adolescent students with hearing impairment: implications for physical education. Medicine (Baltimore). (2020) 99:e21054. 10.1097/MD.0000000000021054 32791677 PMC7387059

[69] Marsh HW Kuyper H Seaton M Parker PD Morin AJS Möller J Dimensional comparison theory: an extension of the internal/external frame of reference effect on academic self-concept formation. Contemp Educ Psychol. (2014) 39:326–41. 10.1016/j.cedpsych.2014.08.003

[70] Owens TJ Samblanet S. Self and self-concept. In: DeLamater J Ward A, Editors. Handbook of Social Psychology. Netherlands, Dordrecht: Springer (2013). pp. 225–49; ISBN 978-94-007-6771-3.

[71] Baena-Extremera A Granero-Gallegos A Ortiz-Camacho M. Quasi-experimental study of the effect of an adventure education programme on classroom satisfaction, physical self-concept and social goals in physical education. Psychol Belg. (2012) 52:369–86. 10.5334/pb-52-4-369

[72] Mouratidis A Lens W Vansteenkiste M. How you provide corrective feedback makes a difference: the motivating role of communicating in an autonomy-supporting way. J Sport Exerc Psychol. (2010) 32:619–37. 10.1123/jsep.32.5.619 20980707

[73] Revuelta L Esnaola I. Clima familiar deportivo y autoconcepto físico en la adolescencia. EJEP. (2011) 4:19. 10.30552/ejep.v4i1.61

[74] Roa García A. La educación emocional, el autoconcepto, la autoestima y su importancia en la infancia. Edetania. (2013):241–57. ISSN: 0214-8560.

[75] Mirabel Alviz M León del Barco B Mendo Lázaro S Iglesias Gallego D. Rol predictivo de la inteligencia emocional y la actividad física sobre el autoconcepto físico en escolares. Sportis Sci J. (2020) 6:308–26. 10.17979/sportis.2020.6.2.5844

[76] Salum-Fares A Aguilar RM Anaya CR. Relevancia de las dimensiones del autoconcepto en estudiantes de escuelas secundarias de ciudad Victoria, tamaulipas, méxico. Rev Elec Psic Izt. (2011) 14:255–72. Available at: https://www.revistas.unam.mx/index.php/repi/article/view/26037 (Accessed January 20, 2024).

[77] Gómez-Mármol A. Relationship between body image self-concept and physical education lessons according to their intensity and enjoyment in secondary education scholars. Eur J Hum Mov. (2013) 31:99–109. ISSN: 0214-0071. Available at: https://www.redalyc.org/articulo.oa?id=274229586007 (Accessed January 20, 2024).

[78] Caballero García MF Alcaraz Muñoz V Alonso Roque JI Yuste Lucas JL. Intensidad emocional en la clase de educación física en función de la Victoria: juegos de cooperación-oposición. REIFOP. (2016) 19:123. 10.6018/reifop.19.3.267291

[79] Amado-Alonso D Mendo-Lázaro S León-del-Barco B Mirabel-Alviz M Iglesias-Gallego D. Multidimensional self-concept in elementary education: sport practice and gender. Sustainability. (2018) 10:2805. 10.3390/su10082805

[80] Prats SB Ortega FZ González MC. Analysis of the levels of self-concept and resilience, in the high school basketball players. Revista de Psicologia del Deporte. (2017) 26:127–32. ISSN: 1132-239X. Available at: https://www.redalyc.org/articulo.oa?id=235150578021 (Accessed January 20, 2024).

[81] Babic MJ Morgan PJ Plotnikoff RC Lonsdale C White RL Lubans DR. Physical activity and physical self-concept in youth: systematic review and meta-analysis. Sports Med. (2014) 44:1589–601. 10.1007/s40279-014-0229-z 25053012

[82] Valero-Valenzuela A Huescar E Núñez JL Conte L Léon J Moreno-Murcia JA. Prediction of adolescent physical self-concept through autonomous motivation and basic psychological needs in Spanish physical education students. Sustainability. (2021) 13:11759. 10.3390/su132111759

[83] Navarro-Patón R Pazos-Couto JM Rodríguez-Fernández JE Arufe-Giraldez V. Measuring physical self-concept of schoolchildren aged 10 to 16 on physical education lessons. JHSE. (2019) 15. 10.14198/jhse.2020.151.01

